# Risk Factors Associated with Leishmaniasis in the Most Affected Provinces by *Leishmania infantum* in Morocco

**DOI:** 10.1155/2020/6948650

**Published:** 2020-06-25

**Authors:** Maryam Hakkour, Asmae Hmamouch, Mohamed Mahmoud El Alem, Abdelhakim Bouyahya, Abdelaali Balahbib, Abdelhak EL Khazraji, Hajiba Fellah, Abderrahim Sadak, Faiza Sebti

**Affiliations:** ^1^Laboratory of Zoology and General Biology, Faculty of Sciences, Mohammed V University in Rabat, Rabat, Morocco; ^2^National Reference Laboratory of Leishmaniasis, National Institute of Hygiene, Rabat, Morocco; ^3^Agronomy and Veterinary Institute Hassan II, Department of Parasitology, Rabat, Morocco; ^4^Laboratory of Microbial Biotechnology, Sciences and Techniques Faculty, Sidi Mohammed Ben Abdellah University, Fez, Morocco; ^5^Laboratory of Human Pathologies Biology, Faculty of Sciences, University Mohammed V, Rabat, Morocco; ^6^Medical Biotechnology Laboratory, Faculty of Medicine and Pharmacy of Rabat, University Mohammed V, Rabat, Morocco

## Abstract

**Background:**

Human leishmaniasis, both visceral and cutaneous, has been reported in Morocco for centuries and constitutes a serious public health problem. However, the evolution of this pathology depends on several factors such as ecological, socioeconomic, and climatic conditions. The risk study of the affected foci is of great value for the control and surveillance of this endemic disease, especially in the provinces where *Leishmania infantum* predominates.

**Methods:**

This study concerned nine provinces located in the extreme and central north of Morocco (Taounate, Taza, Chefchaouen, Al Hoceima, Larache, Tétouane, Tanger-Assilah, M'diq-Fnideq, and Fahs-Anjra Provinces). In this work, leishmaniasis cases (VL and CL) were subjected to an epidemiological study which was performed using a linear regression model to identify the impact as well as the interaction between all predictor variables on the distribution of leishmaniasis in this region.

**Results:**

During the period 1997–2018, a total of 6 128 cases of VL and CL were recorded in the study area. Our results showed that among demographic factors studied, urbanization showed significance for both cutaneous and visceral forms (*P* < 0.05). Regarding the environmental factors, the humidity and the altitude were significant for both CL and VL (*P* < 0.05), while the temperature and the normalized difference vegetation index (NDVI) showed a significance only for VL. Moreover, trends in season of occurrence revealed that wet season (October to April) had a higher incidence of leishmaniasis compared to the dry season (May to September) specifically for CL. As for socioeconomic factors, poverty was the only factor that influences the spread of VL. Finally, the distance from endemic foci showed significance for both VL and LC (*P* < 0.05).

**Conclusion:**

Our study revealed that the risk factor associated with cutaneous and visceral leishmaniasis in northern Morocco could help in the establishment of a prediction program.

## 1. Introduction

Environmental conditions and socioeconomic and demographic factors have a serious impact on human health, particularly on vector-borne infectious diseases including leishmaniasis [1]. In fact, leishmaniasis is an endemic parasitic disease in most tropical regions of the world with approximately two million new human cases reported each year [2]. In Morocco, it is considered among the main endemic disease [3]. The parasite species belonging to the genus *Leishmania* are responsible for different clinical pathologies (visceral and the cutaneous form) according to the localization of these parasites in mammalian tissues [4].

The visceral leishmaniasis (VL) caused by *Leishmania infantum* has been endemic in Morocco for several decades. This disease was essentially limited in the north of Morocco but has shown a remarkable extension over time, and many cases have been observed in other regions [5]. Between 1990 and 2014, the average annual incidence rate was 0.4 VL cases per 100 000 inhabitants [6]. Concerning the cutaneous leishmaniasis (CL), it is caused by three *Leishmania* species: *Leishmania major*, *Leishmania tropica*, and *Leishmania infantum* with more than 3 700 cases reported yearly in this country between 1997 and 2018. The peaks are registered in 2010 and 2018 with, respectively, 8 707 and 9 700 cases [7].

In Morocco, the epidemiological situation of leishmaniasis as well as the distribution of *Leishmania* species varies since the country comprises several geographical areas with different socioeconomic, environmental, and ecological characteristics [8]. In this context, the purpose of this study was to establish epidemiological data on leishmaniasis in the northern region of Morocco known to be moderately affected by CL and highly affected by the VL during the 21-year period (1997–2018) and to determine the impact of environmental fluctuations (temperature, rainfall, humidity, altitude, and NDVI) and the role of demographic factors (population density), socioeconomic (poverty and vulnerability rate), and other indicators (distance to leishmaniasis foci) as risk factors on the propagation of the leishmaniasis in this region.

The epidemiological situation of leishmaniases in the studied provinces was statistically supported in order to identify the various factors responsible for the evolution of this pathology: demographic factors (population density, urbanization, age, and sex); environmental factors (temperature, rainfall, humidity, altitude, NDVI, and seasonality of infection); socioeconomic factors (poverty rate and vulnerability rate), and other factors (distance to leishmaniasis foci). A linear regression model was chosen to study the impact of each factor on all the provinces as well as on each province to illustrate the different modeling possibilities.

## 2. Methods

### 2.1. Study Area

**Figure 1:**
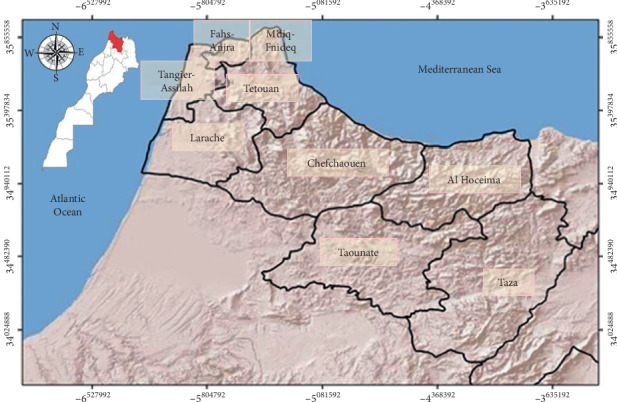
Study area.

This study was established in nine provinces located in the extreme north and central north of Morocco: Taounate, Taza, Chefchaouen, Al Hoceima, Larache, Tetouan, Tanger-Assilah, M'diq-Fnideq, and Fahs-Anjra Provinces (Figure 1).

These zones are part of the north, northwest, and northeast section of the Arc du Rif; they are also known as “Jbala”. Located in the North of Morocco (35° 15′ 00″ North, 5° 56′ 00″ West), they are bordered to the north by the Strait of Gibraltar and the Mediterranean Sea, to the west by the Atlantic Ocean, to the southwest by the region of Rabat-Sale-Kenitra, in the south by the provinces of Sefrou and Moulay Yaacoub, and in the east by the region of the Oriental [8].

On the climatic level, these provinces are characterized by a Mediterranean climate with cold and wet winter. The total population in this study area is approximately 4 446 757 according to the 2014 General Population and Housing Census (RGPH) [9]. Regarding the urbanization rate, it varies between 12.5% recorded in Chefchaouen Province and 100% recorded in Fahs-Anjra Province (Table 1).

### 2.2. Data Collection

A total of 6 128 cases were registered in these provinces of study between 1997 and 2018. Data on human cases were obtained from the Epidemiology and Disease Control Directorate [5]. Environmental variables (temperature, humidity, altitude, and NDVI) were obtained from https://fr.climate-data.org, while normalized difference vegetation index data were collected from the Royal Center for Remote Sensing Space (http://www.crts.gov.ma/bulletins-campagne-agricole).

Data on poverty and the vulnerability rate were obtained from the High Commission for Planning, indicators of poverty and vulnerability in Morocco [10]. However, data on the urbanization rate and population were obtained from the monograph of the Tangier-Tetouan-Al Hoceima and Fez-Meknes regions [11].

According to the High Commission for Planning, the poverty rate is the proportion of individuals (or households) with a low standard of living. Indeed, it is estimated that in 2007, people earning less than 3834 MAD (380€) in urban areas and 3569 MAD (350€) in rural areas in Morocco are considered poor [10]. However, the vulnerability rate to poverty is the proportion of individuals who spent annually 1.5 times more than the threshold of poverty rate. Regarding the urbanization rate, it is the ratio of the urban population to the total population [12].

### 2.3. Statistical Analysis

Statistical analysis was performed using software R version 3.3.3 (http://www.R-project.org). The regression model was applied to evaluate the impact of several factors. The correlation between the total number of leishmaniasis cases and socioeconomic, demographic, and environmental predictors and distance with leishmaniasis foci (in km) was tested using Pearson rank correlation as previously described [13]. The significance level was *P* < 0.05.

## 3. Results

### 3.1. Temporal Distribution of VL and CL Cases between 1997 and 2018 in the Study Area

**Figure 2:**
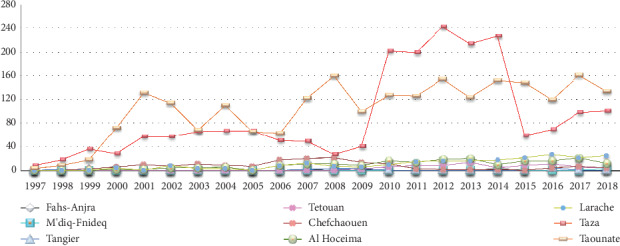
Cutaneous leishmaniasis cases between 1997 and 2018 in the study area.

According to the Moroccan Ministry of Health, a total of 6 128 cases of VL and CL were recorded in the study area. These provinces are known as moderately affected by the CL and strongly affected by the VL compared to the other provinces of the kingdom. Cutaneous form represented 6.34%, the majority of cases were recorded in Taounate Province (45.90%, 2 271/4 947) and Taza Province (39.94%, 1 976/4 947) followed by Larache, Al Hoceima, and Chefchaouen Provinces which recorded 4.54% (225/4 947), 4.08% (202/4 947), and 3.3% (163/4 947), respectively. Concerning the provinces of Tetouan, Tangier-Assilah, Fahs-Anjra, and M'diq-Fnideq, they have recorded 1.53% (76/4 947), 0.34% (17/4 947), 0.22% (11/4 947), and 0.12% (6/4 947) (Figure 2).

**Figure 3:**
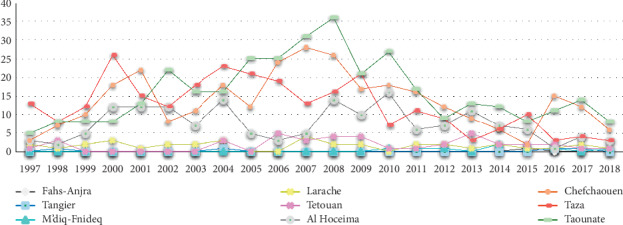
Visceral leishmaniasis cases between 1997 and 2018 in the study area.

Concerning visceral leishmaniasis, it represents 25.33% of reported cases in Morocco and is distributed as follows: 29.88% (353/1 181) in Taounate Province, 25.4% (300/1 181) in Chefchaouen Province, 23.11% (273/1 181) in Taza Province, 14% (165/1 181) in Al Hoceima Province, 3.38% (40/1 181) in Tetouan Province, 3.04% (36/1 181) in Larache Province, 0.67% (8/1 181) in M'diq-Fnideq Province, 0.33% (4/1 181) in Tangier-Assilah Province, and 0.16% (2/1 181) in Fahs-Anjra Province (Figure 3).

### 3.2. Risk Factors Associated with LC and LV

#### 3.2.1. Demographic Factors

Different demographic variables were studied in order to know which one influences the annual number of cases in each province.

*(1) Population Density*. Statistical analysis by linear regression (Table 2) showed that the distribution of leishmaniases does not take into account the number of inhabitants (*P* value > 0.05) with a low coefficient of correlation recorded as well as for CL (*R*^2^ = 0.048) and for VL (*R*^2^ = 0.020).

The distribution of the population in the nine provinces of study varies between 42 914 and 1 136 967 according to the data from the High Commission for Planning [9]. The statistical analysis between 1997 and 2018 by province showed that the number of inhabitants does not influence the number of cases of the CL and the VL except for the province of Tangier-Assilah where a correlation coefficient was 1 and a significant *P* value = 0.003 was detected between the number of cases of VL and the rate of population.

*(2) Urbanization*. In the study provinces, the rate of urbanization varies from one province to another. The analysis of the results showed that the status of the province in terms of urbanization influences the number of cases of CL and VL. Thus, the number of cases increases with the decrease in the rate of urbanization. Since, in the most affected provinces Taounate (2 271 cases of CL and 353 cases of VL) and Taza (1 976 cases of CL and 273 cases of VL), the rate of urbanization was 13.02% and 39.36%, respectively. In the moderately affected provinces Al Hoceima (202 cases of CL and 165 cases of VL), Chefchaouen (136 cases of CL and 300 cases of VL), and Larache (225 cases of CL and 36 cases of VL), the rate of urbanization was around 12.5%, 32.5%, and 53.5%, respectively. In the provinces slightly affected, namely, Tetouan (76 cases of CL and 40 cases of VL), Tangier-Assilah (17 cases of CL and 4 cases of VL), M'diq-Fnideq (6 cases of CL and 8 cases of VL), and Fahs-Anjra (11 cases of CL and 2 cases of VL), it is characterized by the rate of urbanization of 72.31%, 94.30%, 94.30%, and 100%, respectively.

The statistical test gave a correlation coefficient *R*^2^ = 0.33 for CL and *R*^2^ = 0.83 for VL with a *P* value < 0.05. This significant finding revealed that rural provinces were more affected than urban provinces (Table 2).

**Figure 4:**
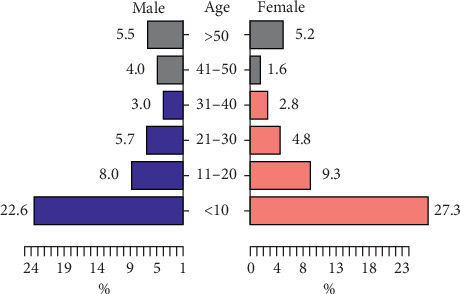
Distribution of leishmaniasis according to age and sex.

*(3) Age and Sex*. The statistical study of the reported cases of leishmaniasis showed that there is no significant difference between the sexes (*χ*^2^ = 0.58, d*f* = 1, *P*=0.44), with a slight predominance of the female sex (51.08% women vs. 48.91% men; sex ratio F/M = 1.04) (Figure 4).

Regarding the distribution of affected ages, all age groups are concerned with leishmaniasis. However, children under 10 years were the most affected with 48% of cases (*χ*^2^ = 1060.8, d*f* = 5, *P* < 2.2*e* − 16).

#### 3.2.2. Environmental Factors

*(1) Temperature*. The statistical study, using linear regression, of the impact of temperature between the provinces (Table 3) showed that the distribution of CL does not take into account the rate of temperature; while for VL, a correlation coefficient of *R*^2^ = 0.48 was calculated with a *P* value = 0.03, so the cases of VL take into account the temperature rate.

However, the analysis of the impact of annual temperature variations on the annual number of cases by province showed that this result was significant only in Al Hoceima (*P* value <0.05, *R*^2^ = 0.32) and Larache Provinces (*P* value <0.05, *R*^2^ = 0.38). On the other hand, the study showed that the number of VL cases takes into consideration the variation of temperature exclusively in Taza Province (*P* value = 0.001, *R*^2^ = 0.41).

*(2) Rainfall*. Regarding the rate of rainfall, the results of the analysis showed that this factor does not impact the distribution for both CL (*P* value = 0.7, *R*^2^ = 0.02) and VL (*P* value = 0.9, *R*^2^ = 0.003) (Table 3).

*(3) Humidity*. The analysis of the impact of humidity showed that this factor influences the distribution of leishmaniases with a significant value detected for both CL (*P* value = 0.002, *R*^2^ = 0.74) and VL (*P* value = 0.001, *R*^2^ = 0.81) (Table 3).

In fact, the provinces reporting the highest number of cases are characterized by a less humid climate compared with other provinces. However, Taounate and Taza Provinces are classified as bioclimatic zones ranging from subhumid to semiarid with an average humidity of 54.4% and 58%, respectively. As for the other provinces with fewer cases (Chefchaouen, Al Hoceima, Larache, Tetouan, Tangier-Assilah, M'diq-Fnideq, and Fahs-Anjra), they are classified in a bioclimatic zone ranging from per-humid to subhumid area with an average humidity of 61%, 72.6%, 73%, 70.3%, 72.2%, 70.3%, and 71.2%, respectively, detected in these provinces.

*(4) Altitude*. The statistical study of the impact of altitude revealed that the number of leishmaniasis cases increases with the increase of the altitude (*P* value <0.05) with a high coefficient of correlation (*R*^2^ = 0.65 for CL) and (*R*^2^ = 0.93 for VL) (Table 3).

*(5) NDVI*. Regarding the vegetation index (NDVI), the analysis of the results showed that only the number of cases of VL takes into account this factor with a low *P* value (=0.0003). However, a high *P* value was calculated for the CL (*P* value = 0.32) which indicates that the latter does not take into account the NDVI (Table 3).

*(6) The Seasonality of Infection*. The trends of the season onset revealed that the incidence of leishmaniasis was higher during the wet season (October to April) compared to the dry season (May to September) specifically for CL (*χ*^2^ = 55.323 d*f* = 1, *P* value = 1.023*e* − 13). For VL, a slight dominance of the wet season was noted (*χ*^2^ = 0.820, d*f* = 1, *P* value = 0.36) (Table 4).

#### 3.2.3. Socioeconomic Factors

*(1) Poverty Rate*. In the study area, the poverty rate varies between 2.9% and 23.4% according to the data from the High Commission for Planning [9]. The analysis of the results, between provinces, showed that the cumulative number of CL cases recorded does not depend on poverty (*P* value = 0.06, *R*^2^ = 0.41). However, the recorded cases of VL takes into account the variation of the poverty rate (*P* value = 0.005, *R*^2^ = 0.69).

By studying the impact of this factor on the distribution of leishmaniasis cases in each province over the years (from 2004 to 2018), it was showed that the latter does not affect the distribution of the CL cases where *P* value was greater than 0.05.

Nevertheless, this factor affects the number of cases of VL in four provinces where the number of cases decreased with the decrease in the poverty rate; these are Chefchaouen (*P* value = 0.01, *R*^2^ = 0.40), Taounate (*P* value = 0.01, *R*^2^ = 0.39), Taza (*P* value = 0.01; *R*^2^ = 0.38), and M'diq-Fnideq (*P* value = 0.04, *R*^2^ = 0.28) (Table 5).

*(2) Vulnerability Rate*. The vulnerability rate varies between 8% and 23.5% in the study area. Statistical analysis by linear regression (Table 5) showed that the distribution of leishmaniasis does not depend on this factor neither for CL (*P* value = 0.06, *R*^2^ = 0.40) nor for VL (*P* value = 0.065, *R*^2^ = 0.42).

#### 3.2.4. Other Factors (Distance to Foci of Leishmaniasis)

The statistical study of the impact of the neighborhood with known foci of leishmaniasis showed that the number of leishmaniasis cases increases with the decrease of the distance (*P* value <0.05) with a high coefficient of correlation recorded for both CL (*R*^2^ = 0.77) and VL (*R*^2^ = 0.71) (Table 6).

## 4. Discussion

This study concerned nine provinces located in the extreme north and central north of Morocco, namely, Taounate, Taza, Chefchaouen, Al Hoceima, Larache, Tetouan, Tanger-Assilah, M'diq-Fnideq, and Fahs-Anjra Provinces.

According to the Moroccan Ministry of Health, 7.63% of the leishmaniasis cases reported were recorded in these provinces (*n* = 6 128/80 299). In terms of type, the cutaneous form represented 6.34% of the cases declared (*n* = 4 947/78 001), while the visceral form represented 51.39% (*n* = 1 181/2 298).

The results of the retrospective study showed that the majority of leishmaniasis cases were recorded in Taounate and Taza Provinces, followed in the decreasing order by Chefchaouen, Al Hoceima, Larache, Tetouan, Tanger-Assilah, M'diq-Fnideq, and Fahs-Anjra provinces. Concerning Taounate and Taza Provinces, they are known by their epidemic past with respect to CL and VL [14–16]. The epidemiological profile in these two provinces was widely described by researchers, who have suggested that the high number of cases can be explained by the fact that the parasite could have been present in rural localities and when it was introduced into periurban areas, and it found conditions conducive to an epidemic, including environmental changes associated with rapid and unplanned development, increased density of man and sandfly, and a decrease in immunity of populations [16].

The epidemiological survey carried out in Taounate Province showed that the number of cases has been increasing since 2001 with peaks recorded in 2008, 2012, 2014, and 2017. However, in Taza Province, a sharp increase has been raised since 2010. This increase was mainly explained by the active screening carried out following the implementation of the response action plan which covered the period from 2010 to 2012. This plan had as objective, on the one hand, the reduction in the incidence of cutaneous leishmaniasis by 50% at the end of 2012 at the level of the 16 main epidemic foci including Taounate and Taza and on the other hand the taking charge of 100% of cases of visceral and cutaneous leishmaniasis.

However, in the other provinces of the extreme north, peaks in the number of CL cases were recorded in three provinces (Larache, Al Hoceima, and Chefchaouen). This number is continuously increasing in the provinces of Larache and Al Hoceima, while in the province of Chefchaouen, a remarkable decrease has been noted. In the rest of the provinces studied, the disease is endemic with a few cases declared annually. Concerning the visceral form, a strong declaration of cases has been recorded particularly in Chefchaouen and Al Hoceima Provinces since the beginning of the census of cases in 1997. Several factors may explain the increase in the number of cases in the provinces of the extreme north. On the one hand, the neighborhood of these provinces with several foci of CL and VL, such as Sidi Kacem [17], Ouazzane [17], Taounate [15], and Taza [16]. On the other hand, through the activities of the population and their movement to unscathed areas [18].

In addition to these epidemiological studies, measures to control this pathology must take into account the parasite cycle since each species of the *Leishmania* genus has its own sandfly species and its own reservoir, and each focus is characterized by the species which circulates as well as the risk factors likely to influence its development. However, molecular investigations recently carried out in these provinces showed the coexistence of *L. tropica* besides *L. infantum* with a predominance of the latter species [19, 20].

Regarding the risk factors associated with CL and VL, several elements influence the dynamics and functioning of the *Leishmania* cycle such as ecological, socioeconomic, and climatic conditions [21]. The risk study of the affected foci is of great use for the control and surveillance of endemic diseases, including leishmaniasis. In our study, the epidemiological situation of leishmaniasis was supported by a statistical analysis in order to identify the various factors such as demographic, environmental, and socioeconomic factors, likely to have a remarkable impact on the evolution of this pathology.

Among the demographic factors studied, the analysis of the results showed that the province's status in terms of urbanization greatly influences the number of CL and VL cases. Indeed, the number of cases increases with the decrease in the urbanization rate. In the most affected provinces (Taounate, Taza, Al Hoceima, and Chefchaouen), the urbanization rate was around 13.02%, 39.36%, 12.5%, and 32.5%, respectively. In the moderately affected province of Larache, the urbanization rate was around 53.5%. As for the weakly affected provinces: Tetouan, Tanger-Assilah, M'diq-Fnideq, and Fahs-Anjra, they had an urbanization rate of around 72.31%, 94.30%, 94.30, and 100%, respectively.

The statistical results showed and confirmed that urbanization affects the spread of leishmaniasis with the exception of Taza Province. In fact, the majority of Taza cases were recorded in rural areas (Oued Amlil, Bouchefaa, Bouhlou, and Bni Frassen). The urban sector of Taza Haut that has accumulated the totality of cases (614 cases) is characterized by its particular geographic location. In fact, it is a very old sector, which is located in a mountainous area; it is surrounded by old cracked and unrestored walls with a nearby river and caves which provide favorable resting places for sandflies.

According to the WHO, the urbanization rate is indicated as a key factor in the increase of leishmaniasis [22]. Transmission of leishmaniasis generally occurs in rural areas [21], where it could be linked to human behavior through the presence of animals and the accumulation of their waste near habitats [23]. Thus, Boussaa et al. have confirmed that this factor has a considerable influence on sandfly populations and therefore on the epidemiology of the disease. The abundance of sandflies seems to decrease with the increase in the urbanization rate and certain potential vectors could disappear [24]. In addition, the movement of populations from neighboring rural households to periurban areas can increase the number of leishmaniasis cases due to poor quality of life and socioeconomic status [25, 26]. These factors constitute favorable conditions for the spread of reservoir hosts and vectors and therefore for the spread of the disease [25].

Regarding the clinical study of leishmaniasis, the age distribution of CL and VL cases showed that no age group was spared from leishmaniasis with a predominance of children aged less than 10 years. This dominance could be explained by the weakness of their immune system and therefore by the inability to defend themselves against infection. In addition, this may also be due to the habits of children who often play near breeding sites, which makes them prone to insect bites [27]. In addition, this study also showed a slight predominance of the male gender with leishmaniasis. This could be explained mainly by the rural character of the provinces where the activities of the population depend closely on the breeding generally practiced by men. The environment in which this type of activity is carried out provides sufficient organic matter favoring the multiplication of sandfly larvae [28]. In addition, this dominance could also be linked to the etiological character of the species *P. perniciosus* which is known to be exophilic [29].

As for environmental factors, leishmaniasis is strongly affected by variations in precipitation, temperature, and humidity. Global warming and soil degradation have a common impact on the epidemiology of leishmaniasis [30]. Indeed, the life cycle of leishmaniasis is sensitive to changes in temperature, rainfall, and humidity. These changes can affect parasites, vectors, and reservoirs by modifying the distribution and influencing survival rates, the population size as well as their dynamic interaction and their territorial expansion [31].

Among climatic factors, temperature is the main factor influencing the spread of leishmaniasis. Indeed, even the smallest variations in temperature can have a profound impact on the development cycle of *Leishmania* promastigotes in sandflies and on their infesting power [32] and thus allow the parasite to be transmitted where the disease was not previously endemic [30]. Another factor that can influence the spread of this parasitosis is the vegetation which also plays an important role in the process of proliferation and growth of sand flies and therefore in the outbreaks of leishmaniasis [33]. The visceral form transmission generally occurs in areas with abundant vegetation [30]. Indeed, Ready reported that deforestation leads to an increase in leishmaniasis [34].

For this, the correlation coefficients between climatic factors and the prevalence of leishmaniasis were calculated. The results obtained showed that humidity, temperature, and precipitation affect the distribution, especially of the visceral form.

On the other hand, a positive correlation was observed between the prevalence of CL and VL and the vicinity with leishmaniasis foci, which remains a major risk responsible for the increase in the number of cases. The provinces having recorded a maximum number of cases are located in the vicinity of the provinces known as foci of leishmaniasis, in particular Taounate and Taza Provinces [15, 19].

## 5. Conclusions

Recently, there are a number of challenges for controlling and preventing leishmaniasis. Our results showed that socioeconomic factors, mainly urbanization, contribute significantly to the maintenance of both CL and VL. However, VL is strongly associated with environmental influences. As a result, in this region, control and prevention strategies must be oriented according to each risk factor studied in order to have a more effective result.

## Figures and Tables

**Table 1:** Population, urbanization rate, and poverty rate by province of the study [9].

Administrative region	Province	Population	Urbanization rate (%)	Poverty rate (%)
Tanger-Tetouan-Al Hoceima	Tanger-Assilah	1 065 601	94.3	2.9
	Fahs-Anjra	76 447	100.0	13.8
	M'diq-Fnideq	209 897	94.3	3.2
	Larache	496 687	53.5	12.2
	Chefchaouen	457 432	12.5	18.8
	Tetouan	550 374	72.3	9.7
	Al Hoceima	399 654	32.5	12.7

Fez-Meknes	Taounate	662 246	13.0	23.4
	Taza	528 419	39.3	15.1

**Table 2:** Results of the linear regression of leishmaniasis in relation to demographic factors.

Factor	Variable		Coefficient		*T*-statistic	*P* value (significance)
			Intercept	Variable		
Demographic	Population density	CL	2.008*e* + 02	7.074*e* − 04	*F*_(1,7)_ = 0.3529	0.5712 (NS)
		VL	9.573*e* + 01	7.197*e* − 05	*F*_(1,7)_ = 0.1395	0.7198 (NS)
	Urbanization	CL	11.000	152.000	*F*_(7,1)_ = 1.527*e* + 04	0.006231 (S)
		VL	345.432	−3.767	*F*_(1,7)_ = 36.140	

NS = not significant; S = significant.

**Table 3:** Results of the linear regression of leishmaniasis in relation to environmental factors.

Factor	Variable		Coefficient		*T*-statistic	*P* value (significance)
			Intercept	Variable		
Environmental	Temperature	CL	9294,5	−493,13	*F*_(1,7)_ = 0.817	0.396 (NS)
		VL	3136.10	−169.44	*F*_(1,7)_ = 6.609	0.037 (S)
	Rainfall	CL	1075.417	−0.8118	*F*_(1,7)_ = 0.1614	0.6999 (NS)
		VL	161.07396	−0.04609	*F*_(1,7)_ = 0.02006	0.8913 (NS)
	Humidity	CL	7764.18	−107.5	*F*_(1,7)_ = 20.83	0.0025 (S)
		VL	1333.9	−17.92	*F*_(1,7)_ = 31.54	0.001 (S)
	Altitude	CL	−62.665	2.3068	*F*_(1,7)_ = 13.13	0.0084 (S)
		VL	14.342	0.4403	*F*_(1,7)_ = 100.8	2.083*e* − 05 (S)
	NDVI	CL	169.00	−114.40	*F*_(2,5)_ = 1.535	0.3202 (NS)
		VL	158.00	−141.60	*F*_(2,5)_ = 107.5	1.3 (S)

NS = not significant; S = significant.

**Table 4:** The onset season of leishmaniasis.

	Season	Percentage (%)	*P* value
CL	Dry	38.70	1.023*e* − 13
	Wet	61.30	
VL	Dry	44.87	0.365
	Wet	55.13	

**Table 5:** Results of the linear regression of leishmaniasis in relation to socioeconomic factors.

Factors	Variable		Coefficient		*T*-statistic	*P* value (significance)
			Intercept	Variable		
Socioeconomic	Poverty rate	CL	−534.68	87.29	*F*_(1,7)_ = 4.99	0.060 (NS)
		VL	−91.537	17.932	*F*_(1,7)_ = 15.7	0.0054 (S)
	Vulnerability rate	CL	−1007.68	99.41	*F*_(1,7)_ = 4.777	0.06 (NS)
		VL	−124.235	16.306	*F*_(1,7)_ = 5.268	0.06 (NS)

NS = not significant; S = significant.

**Table 6:** Results of the linear regression of leishmaniasis in relation to factor of distance to foci of leishmaniasis (in km).

Factors	Variable		Coefficient		*T*-statistic	*P* value (significance)
			Intercept	Variable		
Other indicators	Distance to foci of leishmaniasis (in km)	CL	1732.68	−11.510	*F*_(1,7)_ = 24.7	0.0016 (S)
		VL	311.90	−1.758	*F*_(1,7)_ = 17.57	0.004 (S)

S = significant.

## Data Availability

All data generated during the study are included in this article. More details and precision are available from the author via maryam.hakkour@gmail.com upon reasonable request.
